# School-based health screenings for early detection of pediatric sensory and musculoskeletal conditions: A cross-sectional study

**DOI:** 10.1097/MD.0000000000048903

**Published:** 2026-05-22

**Authors:** Murat Solmaz, Devran Demir

**Affiliations:** a Department of Pediatrics, Batman Iluh State Hospital, Batman, Türkiye; b Department of Pediatrics, Devran Demir Clinics, İzmir, Türkiye.

**Keywords:** early diagnosis, hearing loss, pes planus, school health, scoliosis, screening programs, Turkish children, vision impairments

## Abstract

Vision, hearing, pes planus, and scoliosis are common conditions in school-aged children and can impair learning and long-term health if unrecognized. School-based screening offers a scalable route to early identification and timely referral. We aimed to evaluate the yield of a large provincial school screening program and describe subsequent diagnostic and management outcomes. In this cross-sectional study, we retrospectively evaluated routine school health screenings conducted in Batman, Türkiye, during the 2022 to 2023 academic year. A total of 8433 students (6–17 years) underwent standardized assessments: Snellen visual acuity testing, otoscopy followed by pure-tone audiometry (500–4000 Hz at 20 decibels hearing level), clinical inspection for pes planus, and the Adams forward-bend test. Screen-positive children were referred for confirmatory diagnosis and management. Demographic characteristics, definitive diagnoses, initial treatments, and follow-up plans were summarized descriptively. At least 1 abnormal finding was detected in 1876/8433 (22.3%) students. Screening yields were as follows: vision 695 (8.24%), hearing 308 (3.65%), pes planus 753 (8.93%), and suspected scoliosis 120 (1.42%). Among vision-positive cases, the distribution of refractive errors was myopia 405 (58.3%), astigmatism 224 (32.2%), and hyperopia 66 (9.5%); spectacles were prescribed in 688/695 (99.0%). Hearing-positive cases were unilateral in 202/308 (65.6%) and conductive in 277/308 (89.9%). For pes planus, 698/753 (92.7%) were flexible; 41/753 (5.4%) received orthoses. Of Adams-positive students, 78/120 (65.0%) had radiographic scoliosis (Cobb > 10°), and 45/78 (57.7%) received bracing. School health screening is feasible and low cost, yields clinically actionable findings, and enables early, conservative management of common conditions. Standardized protocols, trained teams, and clear referral pathways can support scale-up and improve child health and educational outcomes. To our knowledge, this is one of the first population-based evaluations from Türkiye linking school screening yields to confirmed diagnoses and initial treatments, with implications for national screening protocols.

## 1. Introduction

Childhood is the most rapid phase of physical, cognitive, and psychosocial development. Chronic conditions that arise or remain undiagnosed during this period can adversely affect learning, school performance, and quality of life, with long-term consequences extending into adulthood.^[1,2]^ Among these, disorders of the sensory systems – particularly refractive errors/myopia and hearing impairment – and musculoskeletal problems such as flexible pes planus and adolescent idiopathic scoliosis (AIS) often progress insidiously and may be underrecognized by families and teachers. The global rise in myopia has been linked to near work and digital screen exposure. Childhood hearing loss is associated with language and educational deficits if not detected early. Most flexible flatfoot is benign but can affect function in a subset of children, and early identification of AIS facilitates timely conservative management (e.g., bracing) and may prevent curve progression.^[3]^

School health screening programs aim to detect asymptomatic disease or risk factors using standardized, low-cost assessments embedded in the education system. Endorsed by the World Health Organization, school-based screening is a pragmatic public health strategy that maximizes population reach and generates actionable, community-level data for referral and timely treatment.^[4]^ Despite these advantages, population-based evidence that links school screening yields to downstream diagnostic confirmation and management pathways remains limited in many settings, including Türkiye.^[5]^

Childhood vision problems are common, with overall rates typically ranging from 5% to 15% in school-aged populations.^[6]^ Refractive errors (myopia, hyperopia, and astigmatism) directly affect classroom performance; untreated myopia can progress, and high myopia increases lifetime risks of retinal detachment, glaucoma, and myopic macular degeneration.^[7]^ Amblyopia most often results from unilateral refractive error or strabismus and may lead to permanent visual loss if not treated during the critical visual-development window in early childhood.^[8]^ In this context, school-based screening provides a pragmatic mechanism for early detection of amblyogenic risk factors and timely referral.

Permanent childhood hearing impairment is estimated at approximately 0.1% to 0.3% globally; when mild–moderate thresholds and unilateral loss are included, prevalence can reach 3% to 5%.^[9]^ Hearing problems in the early school years are linked to language, learning, and psychosocial difficulties if not recognized and treated promptly.^[10]^ Most conductive losses in children are associated with treatable conditions such as otitis media with effusion, underscoring the value of early identification and intervention through standardized pathways.^[11]^

Flexible pes planus is frequent in childhood and is usually asymptomatic; however, painful, rigid, or progressive forms may alter gait and contribute to foot, leg, or back pain in a subset of children.^[12]^ Early recognition enables conservative measures – footwear advice, exercise programs, and orthoses when indicated – to relieve symptoms and prevent functional limitations.^[13]^

AIS – the most common spinal deformity after age 10 years – has a prevalence of approximately 2% to 3%.^[14]^ Because early disease is often asymptomatic, screening programs facilitate timely detection; when initiated during growth, bracing can reduce the risk of curve progression and the need for surgery.^[15]^

We hypothesized that a school-based screening program in a single Turkish province would identify a substantial burden of previously unrecognized vision, hearing, pes planus, and scoliosis among students and that most screen-positive children would receive conservative, clinically meaningful interventions. Therefore, we conducted a population-based evaluation of school health screenings in Batman Province. Specifically, we report the epidemiologic yield of vision, hearing, flexible flatfoot (pes planus), and scoliosis screenings among 8433 school-aged children, and we describe referral outcomes, confirmed diagnoses, and initial treatments. Our goal is to quantify the contribution of school screening to the early identification of conditions that influence educational attainment and long-term health, thereby informing standardization and scale-up of national screening protocols.

## 2. Methods

### 2.1. Study population and ethics

This cross-sectional study included school-aged children who underwent routine school health screening in Batman Province, Türkiye, during the 2022 to 2023 academic year. The screening program was organized by the Provincial Health Directorate in collaboration with the Provincial Directorate of National Education and targeted grades within primary and secondary schools.

All students in participating schools who were present on the screening days and had complete identifiers were eligible. Children with missing identifiers or incomplete screening records for a given outcome were excluded from the analyses of that outcome. Because this was an evaluation of a province-wide program, no a priori sample size calculation was performed; instead, all eligible students screened during the academic year were included, thereby maximizing precision.

The study protocol was approved by the Non-Interventional Research Ethics Committee of Batman Training and Research Hospital (Protocol No. 433, September 30, 2025). Written administrative permission was obtained from the Provincial Directorate of National Education. Because anonymized program data were used, individual informed consent was waived in accordance with institutional policy and national regulations.

### 2.2. Screening protocols

- Vision screening: Each eye was tested separately using a Snellen chart at a standard distance. Visual acuity of ≤6/9 (20/30) in either eye was considered abnormal and prompted referral to the ophthalmology clinic for comprehensive examination and management.
- Hearing screening: Bilateral otoscopy was followed by pure-tone sweep audiometry at 500, 1000, 2000, and 4000 Hz at 20 decibels hearing level. Lack of response at 1 or more frequencies in either ear was deemed screen-positive and triggered referral to audiology. Right and left ears were recorded separately.
- Flatfoot (pes planus) screening: Medial longitudinal arch collapse on weight-bearing inspection (with loss of medial arch support/valgus heel) was considered suspicious; such cases were referred to orthopedics and traumatology for confirmation and counseling.
- Scoliosis screening: The Adams forward-bend test was performed by trained staff. Trunk asymmetry, scapular prominence, or lumbar line discrepancy constituted a positive screen and prompted referral to orthopedics for radiographic evaluation and further management.

### 2.3. Data collection and statistical analysis

For screen-positive children, a structured, 1-page form was used to capture information in a standardized manner. The form included sections to be completed at the time of screening (demographic characteristics, school and grade, screening result, and preliminary impression) and at the referral clinic visit (family history in first-degree relatives, comorbidities such as diabetes or rheumatologic disease, relevant infection history including recurrent otitis media, definitive diagnosis, initial treatment, and planned follow-up). Fields were primarily tick boxes with limited free-text entries to facilitate routine use.

At referral clinics, definitive diagnoses, initial treatments (e.g., glasses or occlusion therapy for vision, medical therapy or ventilation tubes for conductive hearing loss, and orthoses or bracing), and follow-up plans were recorded on the same form. Data were entered into SPSS version 25.0 (IBM Corp.) for analysis.

Descriptive statistics were reported as n (%) for categorical variables and mean ± standard deviation for continuous variables. Group comparisons for categorical outcomes used two-sided chi-square tests. Statistical significance was set at α = 0.05 (*P* < .05). For each variable, analyses were based on all available cases with non-missing data; no imputation was performed, and complete-case analysis was used for the affected variables.

Although we report outcomes among referred children, this analysis is cross-sectional because it summarizes program data from a single screening cycle and the first referral visits within the same academic year, rather than long-term longitudinal follow-up.

## 3. Results

### 3.1. Overall findings

A total of 8433 students were screened; 4122 (48.9%) were female, and 4311 (51.1%) were male. The mean age was 11.4 ± 3.2 years (range, 6–17 years). At least 1 abnormal finding was detected in 1876/8433 (22.3%) students, and 143/8433 (1.7%) had multiple pathologies (7.6% among those with any abnormality; Table 1).

**Table 1:** Prevalence of abnormal screening findings among 8433 students.

Scan type	Number of abnormal findings (n)	Percentage (%)
Visual impairment	695	8.24
Hearing loss	308	3.65
Flat foot	753	8.93
Suspected scoliosis	120	1.42
Total	1876	22.3

### 3.2. Vision outcomes

Among the 695 students with abnormal vision screening results, 368 (52.9%) were male, and 327 (47.1%) were female. The distribution of definitive ophthalmic diagnoses and family history is summarized in Table 2. Spectacles were prescribed for 688/695 (99.0%) cases; occlusion therapy was initiated in 7/695 (1.0%; not mutually exclusive with spectacles).

**Table 2:** Type of visual impairment and family history among screen-positive students (n = 695).

Type of visual impairment	Number (n)	Percentage (%)	Those with a family history n (%)
Myopia	405	58.3	172 (42.5)
Astigmatism	224	32.2	78 (34.8)
Hyperopia	66	9.5	19 (28.8)
Total	695	100	269 (38.7)

### 3.3. Hearing outcomes

Among the 308 students with abnormal hearing screening results, 165 (53.6%) were male, and 143 (46.4%) were female. Characteristics of hearing loss and associated factors are shown in Table 3.

**Table 3:** Characteristics of hearing loss among screen-positive students (n = 308).

Feature	Number (n)	Percentage (%)
Unilateral loss	202	65.6
Double loss	106	34.4
Transmission type loss	277	89.9
Sensorineural hearing loss	31	10.1
History of otitis media	127	41.2

Following audiological evaluation, medical treatment (decongestants, antibiotics) and/or ventilation tube placement was recommended for 243/277 (87.7%) students with conductive hearing loss, whereas hearing aids were deemed appropriate for 28/31 (90.3%) students with sensorineural hearing loss.

### 3.4. Pes planus and scoliosis outcomes

#### 3.4.1. Pes planus

Of the 753 screen-positive cases, 698 (92.7%) were diagnosed with flexible pes planus. Overall, 712/753 (94.6%) were managed with observation and footwear or exercise advice, whereas 41/753 (5.4%) received orthoses; family history was present in 139/753 (18.5%).

#### 3.4.2. Scoliosis

Of the 120 students with a positive Adams test result, 78 (65.0%) were radiographically confirmed with a Cobb angle > 10°. Among confirmed cases, 62/78 (79.5%) were female. Curve patterns were thoracic in 45/78 (57.7%), thoracolumbar in 28/78 (35.9%), and lumbar in 5/78 (6.4%). Management included observation in 29/78 (37.2%), bracing in 45/78 (57.7%), and referral for surgical evaluation in 4/78 (5.1%) for curves >40°.

## 4. Discussion

Our findings underscore the value of school-based health screening for the early identification of conditions that can influence learning and long-term health. Detecting ≥1 abnormality in 22.3% of students supports the premise that population-level screening can uncover a substantial pool of otherwise unrecognized needs and facilitate timely referral and management.

### 4.1. Vision

The proportion of students with abnormal vision screening results (8.24%) aligns with reports that childhood vision problems commonly affect approximately 5% to 15% of school-aged populations.^[6]^ Consistent with global trends, myopia was the most frequent diagnosis, a pattern plausibly related to increased near work and digital screen exposure.^[16]^ The observed family history rate (38.7%) reinforces the role of genetic predisposition and justifies closer surveillance of at-risk children. Nearly universal spectacle prescription (99%) indicates that screening translated into actionable care, which is known to mitigate the academic and psychosocial consequences of uncorrected refractive error.

### 4.2. Hearing

Abnormal hearing screening results in 3.65% of students are within the range expected when mild to moderate and unilateral losses are considered and within the range reported in international studies when unilateral and mild hearing losses are included.^[9,11,17]^ The predominance of conductive hearing loss (89.9%), together with a notable history of otitis media, highlights an opportunity for preventable and treatable pathology if identified early through standardized pathways. Early detection is important because childhood hearing loss – especially when unilateral or mild – can still be associated with language, educational, and psychosocial difficulties if unrecognized.

### 4.3. Pes planus

Pes planus (8.93%) was the most frequent single category in our cohort. Most cases were flexible and asymptomatic, in keeping with the pediatric literature, which cautions against overmedicalization while endorsing conservative measures (footwear advice, exercise, and orthoses when indicated) for symptomatic children.^[18]^ Rates in our sample are broadly consistent with reports from Turkish pediatric populations.^[13]^

### 4.4. Scoliosis

Screening for AIS is valuable because early identification during growth enables conservative management and may prevent curve progression to surgical thresholds. Yilmaz and Erkan’s systematic review also confirms the effectiveness of school screening programs for the early detection of scoliosis.^[19]^ In our study, 65% of Adams-test-positive screening results were radiographically confirmed (Cobb angle > 10°), reflecting a meaningful diagnostic yield in a real-world school program. However, the Adams test is known to generate some false-positive results, particularly in children with postural asymmetry, which underscores the importance of standardized training and clear radiographic referral criteria.^[14,15]^ The high proportion of bracing (57.7%) among confirmed cases is aligned with contemporary International Society on Scoliosis Orthopaedic and Rehabilitation Treatment recommendations favoring observation, exercises, and bracing for appropriate curves during growth.^[20,21]^ These findings suggest that organized screening can shift treatment toward effective nonsurgical options and may reduce downstream surgical burden.

## 5. Data limitations and future research directions

### 5.1. Limitations

Several limitations should be acknowledged. First, this was a single-province study, which may limit generalizability to other regions with different sociodemographic or health system characteristics. Second, we did not estimate test accuracy (sensitivity, specificity, positive and negative predictive values) or inter-rater reliability of individual screening maneuvers against gold-standard assessments in the entire screened population, particularly for the Adams test, which is susceptible to false-positive results. Third, although we used routine program data from a large cohort, we did not stratify prevalence estimates by age or socioeconomic status; such analyses require more detailed individual-level information and will be valuable in future work. Finally, residual confounding by unmeasured factors (e.g., near-work exposure, screen time, and detailed family socioeconomic indicators) cannot be excluded.

### 5.2. Future research directions

In this school-based screening program encompassing 8433 students, 22.3% had at least 1 abnormal finding across vision, hearing, pes planus, and scoliosis – conditions for which effective, low-cost interventions are available. Screening was feasible and translated into care (e.g., spectacles in 99% of vision-positive cases; bracing in 57.7% of radiographically confirmed scoliosis), underscoring its practical value for early identification and management.

Nationwide implementation should adopt standardized protocols, competency-based training, and quality assurance measures (calibration and inter-rater checks), supported by clear referral and feedback pathways. Programs should be monitored with predefined indicators – coverage, positivity, positive predictive value, time to care, and treatment uptake – to optimize performance and minimize false-positive and false-negative results. Future work should assess cost-effectiveness, long-term educational and health outcomes, and equity of access across regions to inform sustainable scale-up.

## 6. Conclusion

In this school-based screening program encompassing 8433 students, 22.3% had at least 1 abnormal finding across vision, hearing, pes planus, and scoliosis – conditions for which effective, low-cost interventions are available. Screening was feasible and translated into care (e.g., spectacles in 99.0% of vision-positive cases; bracing in 57.7% of radiographically confirmed scoliosis), underscoring its practical value for early identification and management.

Nationwide implementation should adopt standardized protocols, competency-based training, and quality assurance (calibration, inter-rater checks), supported by clear referral and feedback pathways. Programs should be monitored with predefined indicators – coverage, positivity, positive predictive value, time to care, and treatment uptake – to optimize performance and minimize false-positive and false-negative results.

Future work should assess cost-effectiveness, long-term educational and health outcomes, and equity of access across regions to inform sustainable scale-up.

## Author contributions

**Conceptualization:** Murat Solmaz, Devran Demir.

**Methodology:** Murat Solmaz, Devran Demir.

**Data curation:** Murat Solmaz, Devran Demir.

**Funding acquisition:** Murat Solmaz.

**Investigation:** Murat Solmaz, Devran Demir.

**Project administration:** Murat Solmaz.

**Formal analysis:** Devran Demir.

**Resources:** Devran Demir.

**Software:** Devran Demir.

**Supervision:** Devran Demir.

**Visualization:** Devran Demir.

**Writing – original draft:** Murat Solmaz.

**Writing – review & editing:** Murat Solmaz, Devran Demir.

## References

[1] Hu N Fardell J Wakefield CE. School academic performance of children hospitalised with a chronic condition. Arch Dis Child. 2022;107:289–96. 34475105 10.1136/archdischild-2020-321285 PMC8862027

[2] Forrest CB Bevans KB Riley AW Crespo R Louis TA. School outcomes of children with special health care needs. Pediatrics. 2011;128:303–12. 21788226 10.1542/peds.2010-3347 PMC3387854

[3] Patton GC Sawyer SM Santelli JS. Our future: a Lancet commission on adolescent health and wellbeing. Lancet (Lond). 2016;387:2423–78. 10.1016/S0140-6736(16)00579-1 PMC5832967 27174304

[4] World Health Organization. School Health Services. WHO Press; 2021. https://www.who.int/publications/i/item/9789240029392.

[5] Lear JG. Health at school: a hidden health care system emerges from the shadows. Health Aff (Project Hope). 2007;26:409–19. 10.1377/hlthaff.26.2.409 17339668

[6] Holden BA Fricke TR Wilson DA. Global prevalence of myopia and high myopia and temporal trends from 2000 through 2050. Ophthalmology. 2016;123:1036–42. 26875007 10.1016/j.ophtha.2016.01.006

[7] Morgan IG Ohno-Matsui K Saw S-M. Myopia. Lancet (Lond). 2012;379:1739–48. 10.1016/S0140-6736(12)60272-4 22559900

[8] Webber AL Wood J. Amblyopia: prevalence, natural history, functional effects and treatment. Clin Exp Optom. 2005;88:365–75. 16329744 10.1111/j.1444-0938.2005.tb05102.x

[9] Olusanya BO Neumann KJ Saunders JE. The global burden of disabling hearing impairment: a call to action. Bull World Health Organ. 2014;92:367–73. 24839326 10.2471/BLT.13.128728 PMC4007124

[10] Wake M Poulakis Z Hughes EK Carey-Sargeant C Rickards FW. Hearing impairment: a population study of age at diagnosis, severity, and language outcomes at 7-8 years. Arch Dis Child. 2005;90:238–44. 15723906 10.1136/adc.2003.039354 PMC1720307

[11] Rosenfeld RM Shin JJ Schwartz SR. Clinical practice guideline: otitis media with effusion (update). Otolaryngol Head Neck Surg. 2016;154(1 Suppl):S1–S41. 26832942 10.1177/0194599815623467

[12] Moore J Tafti D. Pes planus. In: StatPearls. StatPearls Publishing; 2026. 28613553

[13] Ugras AA. İstanbul school-age children hand and foot anomalies study. https://izlik.org/JA65UM74ST.

[14] Weinstein SL Dolan LA Cheng JCY Danielsson A Morcuende JA. Adolescent idiopathic scoliosis. Lancet (Lond). 2008;371:1527–37. 10.1016/S0140-6736(08)60658-3 18456103

[15] Negrini S Donzelli S Aulisa AG. 2016 SOSORT guidelines: orthopaedic and rehabilitation treatment of idiopathic scoliosis during growth. Scoliosis Spinal Disord. 2018;13:10. 29435499 10.1186/s13013-017-0145-8 PMC5795289

[16] Lanca C Saw S-M. The association between digital screen time and myopia: a systematic revie. Ophthalmic Physiol Opt. 2020;40:216–29. 31943280 10.1111/opo.12657

[17] Kaplama ME Ak S. The results of hearing screening in refugee school children living in Şanliurfa/Turkey and the related risk factors. Int J Pediatr Otorhinolaryngol. 1100;134:41. 10.1016/j.ijporl.2020.110041 32289664

[18] Evans AM. The flat-footed child: to treat or not to treat: what is the clinician to do? J Am Podiatr Med Assoc. 2008;98:386–93. 18820042 10.7547/0980386

[19] Dunn J Henrikson NB Morrison CC Blasi PR Nguyen M Lin JS. Screening for adolescent idiopathic scoliosis: evidence report and systematic review for the US preventive services task force. JAMA. 2018;319:173–87. 29318283 10.1001/jama.2017.11669 PMC13480065

[20] Hresko MT. Clinical practice. Idiopathic scoliosis in adolescents. N Engl J Med. 2013;368:834–41. 23445094 10.1056/NEJMcp1209063

[21] Fong DYT Lee CF Cheung KMC. A meta-analysis of the clinical effectiveness of school scoliosis screening. Spine. 2010;35:1061–71. 20393399 10.1097/BRS.0b013e3181bcc835

